# Tunneling nanotube formation is stimulated by hypoxia in ovarian cancer cells

**DOI:** 10.18632/oncotarget.9504

**Published:** 2016-05-20

**Authors:** Snider Desir, Elizabeth L. Dickson, Rachel I. Vogel, Venugopal Thayanithy, Phillip Wong, Deanna Teoh, Melissa A. Geller, Clifford J. Steer, Subbaya Subramanian, Emil Lou

**Affiliations:** ^1^ Integrative Biology and Physiology Program, University of Minnesota, Minneapolis, MN, USA; ^2^ Department of Medicine, Division of Hematology, Oncology and Transplantation, University of Minnesota, Minneapolis, MN, USA; ^3^ Department of Obstetrics and Gynecology, Division of Gynecologic Oncology, University of Minnesota, Minneapolis, MN, USA; ^4^ Biostatistics and Bioinformatics Core, Masonic Cancer Center, University of Minnesota, Minneapolis, MN, USA; ^5^ Departments of Medicine and Genetics, Cell Biology and Development, University of Minnesota, Minneapolis, MN, USA; ^6^ Department of Surgery, Division of Basic Science and Translational Research, University of Minnesota, Minneapolis, MN, USA

**Keywords:** tunneling nanotubes, intercellular communication, hypoxia, chemoresistance, mTOR

## Abstract

In this study, we demonstrated that hypoxic conditions stimulated an increase in tunneling nanotube (TNT) formation in chemoresistant ovarian cancer cells (SKOV3, C200). We found that suppressing the mTOR pathway using either everolimus or metformin led to suppression of TNT formation *in vitro*, verifying TNTs as a potential target for cancer-directed therapy. Additionally, TNT formation was detected in co-cultures including between platinum-resistant SKOV3 cells, between SKOV3 cells and platinum-chemosensitive A2780 cells, and between SKOV3 cells cultured with benign ovarian epithelial (IOSE) cells; these findings indicate that TNTs are novel conduits for malignant cell interactions and tumor cell interactions with other cells in the microenvironment. When chemoresistant C200 and parent chemosensitive A2780 cells were co-cultured, chemoresistant cells displayed a higher likelihood of TNT formation to each other than to chemosensitive malignant or benign epithelial cells. Hypoxia-induced TNT formation represents a potential mechanism for intercellular communication in ovarian cancer and other forms of invasive refractory cancers.

## INTRODUCTION

Ovarian cancer is the most fatal gynecologic cancer in the United States [1]. Platinum-based chemotherapy is the primary treatment for ovarian cancer; however, resistance to chemotherapy continues to be a major clinical problem [2]. The molecular mechanisms of chemotherapy resistance remain unclear. It is widely accepted that chemoresistance emerges as a result of mutations in key regulatory genes with cells passing these genetic mutations via vertical transmission to daughter cells through mitotic division and clonal expansion. However, horizontal (cell-to-cell) transmission of regulatory factors via channels of cellular communication could also be responsible for the development of chemotherapy resistance. This concept was, in fact, proposed as a model of chemoresistance in ovarian cancer over 20 years ago [3].

Modes of intercellular transport that have been well-studied include chemical signaling via cytokines or other diffusible signals, packaging of signals in membrane-enclosed vesicles such as exosomes or microvesicles, and connexin-based gap junctions connecting adjacent cells [4, 5]. Tunneling nanotubes (TNTs) have recently been characterized as cellular conduits of intercellular communication increasingly studied for their role in mediating long-range transport of cellular cargo via direct cell-to-cell contact [6–14]. TNTs are thin, membrane-lined, actin-based conduits that can form connections between cells as far as 100–200 μm apart and transport cellular cargo (including mitochondria and microRNAs) between cells [9, 10, 12]. They are distinguished from other actin-based cellular extensions by their smaller diameter (50–800 nm) and non-adherence to the substratum when cultured *in vitro* [9, 10, 14, 15]. We have successfully imaged nanotube protrusions in resected malignant mesothelioma and pulmonary adenocarcinoma tumors from human patients and between primary malignant cells derived from pleural effusions and ascites [10, 16]. Using confocal microscopy, we also recently reported the presence of TNTs in osteosarcoma resected from mice and also in ovarian tumors surgically resected from human patients [12]. These findings support the potential *in vivo* role of TNTs for human malignancies, including ovarian cancer.

Elucidating new mechanisms of intercellular communication may provide an innovative approach to evaluating the development of chemoresistance. We recently demonstrated that microRNAs (miRNAs), including those differentially expressed in chemoresistant cancers, can be transported via TNTs between malignant ovarian cells and malignant and stromal cells [12]. However, the role of TNTs in cancer pathobiology remains unclear. Here, we investigate TNTs as a novel mechanism for development of drug resistance by assessing TNT formation among chemoresistant and chemosensitive ovarian cancer cell lines under normoxic and hypoxic conditions and the role of TNTs in facilitating intercellular transport of cytotoxic drugs from drug-resistant to drug-sensitive cancer cells.

## RESULTS

### Examination and quantification of TNTs in malignant chemoresistant ovarian cell lines

**Figure 1:**
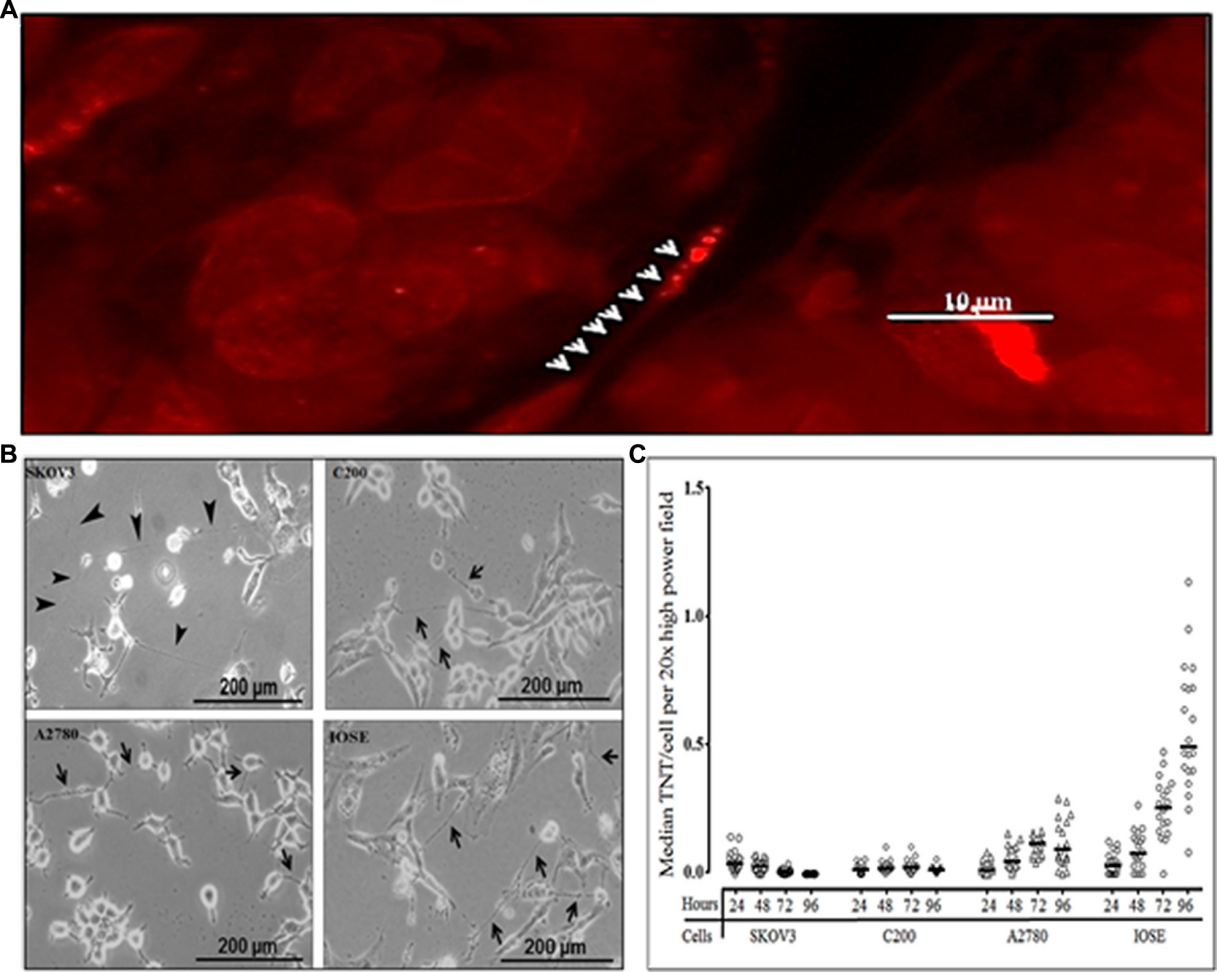
Differing patterns of TNT formation among malignant (chemoresistant and chemosensitive) and also benign ovarian cells (**A**) Representative confocal microscopy image of a TNT within an intact human malignant ovarian tumor (adenocarcinoma). Arrowheads indicate mitochondria within a TNT stained with MitoTracker orange-fluorescent dye. (**B**) Representative phase contrast microscopy images of TNTs connecting the cisplatin- and doxorubicin-resistant SKOV3 ovarian cancer cells; platinum-resistant C200 cells, and their parent chemosensitive cell line A2780; and a benign ovarian epithelial cell line (IOSE). (**C**) Quantification of TNTs/cell per field in cultures of chemoresistant, chemosensitive, and benign ovarian epithelial cell lines across replicates over four days plotted and summarized using the median (line). An Olympus IX70 inverted microscope with 20× objective lens was used to visualize and count the number of TNTs and cells in 10 randomly chosen fields. This experiment was performed in duplicate.

Using confocal imaging, we had previously identified TNT-like structures in malignant ovarian tumors resected from human patients, supporting our hypothesis that TNTs are physiologically relevant cellular structures in this form of cancer [12]; a representative example is shown in Figure 1A. Using inverted microscopic imaging, we identified TNT formation among malignant ovarian cell lines (chemoresistant and chemosensitive) and benign ovarian epithelial cells *in vitro* [10, 12] (Figure 1B). We had previously demonstrated that TNTs form reliably at a quantifiably higher rate when cultured under conditions of metabolic stress, specifically in a low-serum (2.5% FCS), hyperglycemic (50 mM), acidified (pH 6.6) “TNT medium” [10]. We hypothesized that there are differences in the rate of TNT formation between chemoresistant and chemosensitive cells. To address this hypothesis, we sought to quantify the degree of TNT formation the only currently available matched platinum-resistant/sensitive ovarian cancer cell lines, and thus we used them in our study. We cultured each cell line in TNT medium using a predetermined number of sub-confluent cells to allow for optimal TNT formation [10]. We then quantified the number of TNTs and cells per high-power field at 24, 48, 72, and 96 hours (Figure 1C). To account for differences in the rate of cellular proliferation among cell lines, we calculated the average number of TNTs per cell (TNTs/cell). These data were not normally distributed and therefore the raw values are presented and summarized using the median. Interestingly, while the median number of cells per high-power field was significantly higher among chemoresistant cell lines (C200 and SKOV3; Supplementary Figure 1; Supplementary Table 1), the overall rate of TNT formation was greater for the IOSE cell line when reported as TNTs/cell, due to the low proliferation rate of IOSE (Supplementary Table 2). Conversely, for highly proliferative cells that produce few TNTs, the median number TNTs/cell produced a low “TNT index.” Interestingly, TNT formation occurred to a higher degree among the chemosensitive cell line A2780 as compared to chemoresistant cell lines, even accounting for differences in cell proliferation.

### Hypoxic conditions increase TNT formation between chemoresistant ovarian cancer cells

**Figure 2:**
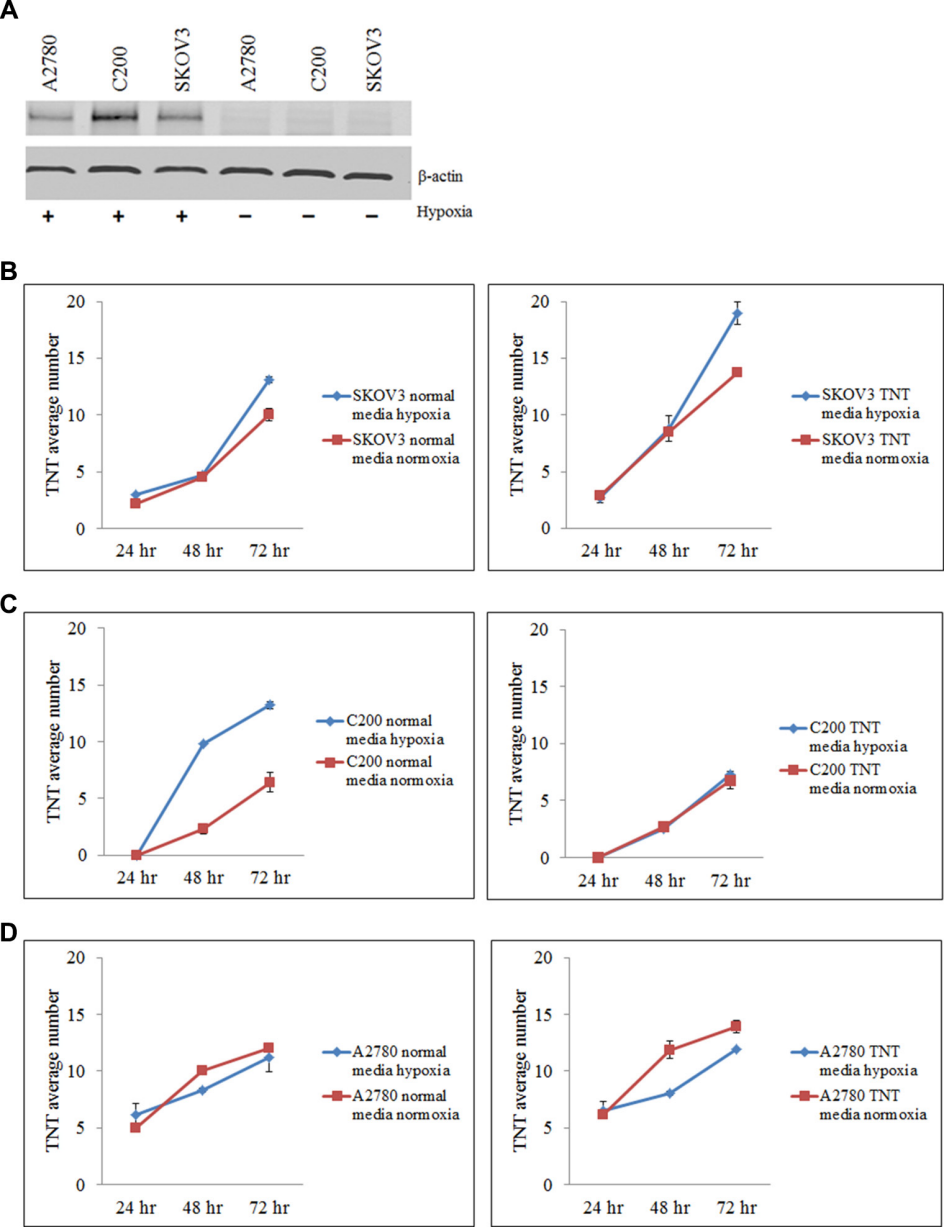
Hypoxia induces HIF-1α expression and TNT formation in ovarian cancer cell lines (**A**) Quantitative Western blot analysis shows increased expression of HIF-1α in SKOV3, C200, and A2780 cells cultured under hypoxic conditions. (**B–D**) Number of TNTs (mean ± standard deviation) in chemoresistant SKOV3 (B) and C200 (C) cells and chemosensitive A2780 (D) cells cultured in standard and TNT media under normoxic (left panel) or hypoxic (right panel) conditions. For all experiments, the number of TNTs in 10 high-power fields (hpf) were counted and averaged. This experiment was performed in duplicate.

TNTs are known to be upregulated under conditions of metabolic stress, including exposure to hydrogen peroxide, serum deprivation, and hyperglycemia [10, 17, 18]. We hypothesized that TNTs would also be induced under conditions of environmental stress characteristic of the tumor microenvironment such as hypoxia. Hypoxia is a hallmark of aggressively proliferating malignant tumors, and has been implicated in the development of chemoresistance [19, 20]. The lack of adequate oxygen in the tumor microenvironment triggers a stress response at the molecular and cellular levels, leading to increased invasiveness and resistance to drug therapy [20–22]. Expression of hypoxia inducible factor-1α (HIF-1α) in mammalian cells is induced as part of the systemic response to low oxygen levels and plays a key role in maintaining cellular homeostasis. As such, HIF-1α can serve as an effective molecular marker of hypoxia. Thus, we initially determined whether hypoxia induces TNT formation in chemoresistant ovarian cancer cells (SKOV3 and C200 cell lines); we also assessed the effect on chemosensitive cells (A2780). To confirm that oxygen deprivation in our culture system induced hypoxia, we examined HIF-1α expression in these cells when cultured in either hypoxic or normoxic conditions for up to 24 hours. We found that HIF-1α expression in A2780, C200, and SKOV3 cells was respectively 6, 10, and 11 fold higher when cultured in hypoxic conditions as compared to normoxic conditions (Figure 2A).

We next determined the effects of hypoxia on TNT formation and cell proliferation in several lines of chemoresistant and chemosensitive ovarian cancer cells. Ovarian cancer cell lines A2780, C200, and SKOV3 were cultured in standard medium conditions (10% fetal calf serum/FCS in RPMI-1640) or TNT medium and under normoxic or hypoxic conditions. We quantitated the number of TNTs connecting cells cultured in hypoxic vs. normoxic conditions. When cells were cultured in standard passage medium under hypoxic conditions, chemoresistant SKOV3 cells demonstrated an increase in TNT numbers by 72 hours as compared to cells cultured in normoxic conditions (Figure 2B, left panel). Similarly, chemoresistant C200 cells also developed more TNTs under hypoxic conditions by 48 and 72 hours (Figure 2C, left panel). No differences were observed in TNT numbers among A2780 cells cultured in hypoxic or normoxic conditions at any timepoint using standard passage medium (Figure 2D, left panel). When cells were cultured in TNT medium, SKOV3 cells (Figure 2B, right panel) and chemoresistant C200 cells (Figure 2C, right panel) again demonstrated a higher rate of TNT formation in hypoxia by 48 and 72 hours, respectively. Notably, chemosensitive A2780 cells formed fewer TNTs under hypoxic conditions by 48 and 72 hours as compared with cells cultured in normoxia (Figure 2D, right panel). From these experiments, we concluded that hypoxia appears to induce TNT formation under metabolic conditions that are already favorable to drug-resistant cell activity.

To account for differences in cellular proliferation under these conditions, we quantified cell number and related changes as well by comparing chemoresistant C200 cells and their parent chemosensitive cell line A2780. In standard culture medium, A2780 cells proliferated at a lower rate under hypoxic conditions than normoxic conditions (Supplementary Figure 2A, left panel). Chemoresistant C200 cells also demonstrated a lower rate of cell proliferation when grown in standard medium and exposed to hypoxia (Supplementary Figure 2B, left panel). Notably, when cells were cultured in TNT medium and subjected to hypoxic conditions, C200 cells proliferated at noticeably higher rates under hypoxic conditions as compared to normoxic conditions in TNT medium (Supplementary Figure 2B, right panel); however, this rate of increase was not proportional to the increased rate of formation of TNTs/cell in the C200 cell line. A2780 cells, however, remained less proliferative in this setting (Supplementary Figure 2A, right panel). Similarly, SKOV3 cells proliferated at a much lower rate in hypoxic conditions as compared to normoxia when cultured in passage culture medium (Supplementary Figure 2C, left panel). However, when cultured in TNT medium, there was very little difference in cell proliferation under hypoxic or normoxic conditions (Supplementary Figure 2C, right panel). These experiments demonstrated that hypoxia induced differences in cellular proliferation among chemoresistant and chemosensitive cell lines. Taken together, our data indicate that increased TNT numbers among chemoresistant (C200 and SKOV3) ovarian cancer cells in a hypoxic environment is not due solely to changes in cell proliferation.

### TNTs form between chemoresistant and chemosensitive ovarian cancer cells *in vitro*

**Figure 3:**
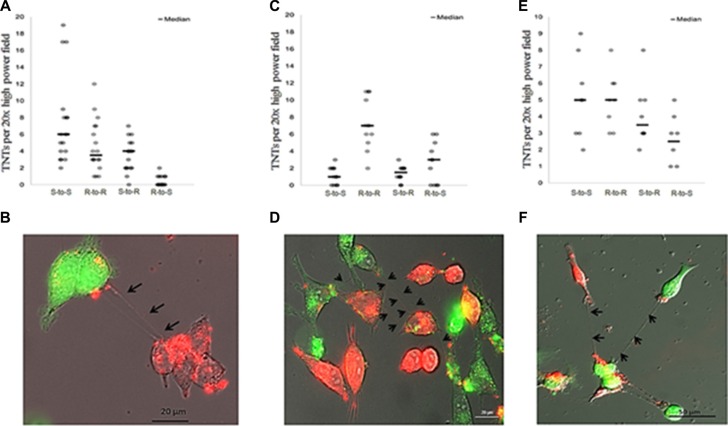
TNT formation between chemoresistant and chemosensitive ovarian cancer cell lines and between chemoresistant ovarian cancer and benign epithelial ovarian cells (**A**) The number of TNTs in co-cultures of chemoresistant SKOV3 (R) and chemosensitive A2780 (S) cells across replicates and summarized using the median (line). (**B**) Representative Zeiss Axio widefield fluorescence microscopy images of TNTs forming among DiI red-labeled A2780 (chemosensitive) and GFP (green)-labeled SKOV3 (cisplatin, adriamycin resistant) cell lines. (**C**) The number of TNTs in co-cultures of chemoresistant C200 (R) cells and chemosensitive A2780 (S) cells across replicates and summarized using the median (line). (**D**) Representative microscopy image of TNTs forming among DiI red-labeled A2780 (chemosensitive) and DiO green-labeled C200 cell lines. (**E**) The number of TNTs in co-cultures of chemoresistant SKOV3 (R) cells and normal ovarian epithelial IOSE (S) cells across replicates and summarized using the median (line). (**F**) Representative microscopy image of TNTs forming among GFP green-labeled SKOV3 cells and DiI red-labeled IOSE cells. For all images in this figure, cells were counted per 20× high power field during a 24-hour period at 15 minute intervals.

Having confirmed that malignant ovarian cancer cells derived from commonly studied cell lines formed TNTs *in vitro*, we next sought to determine whether chemosensitive A2780 cells establish cell-to-cell contact with chemoresistant C200 or SKOV3 cells via TNTs. To distinguish the two cell populations, we either stained cells with fluorescent lipophilic dyes, using DiO (C200, green) and DiI (A2780, red), or used SKOV3 cells transduced with a GFP-expressing lentiviral vector. Under normoxic culture conditions, we reproducibly observed *de novo* formation of TNTs between ovarian chemoresistant and chemosensitive cell lines. We co-cultured these cells in a 1:1 ratio using our TNT growth-promoting conditions (TNT medium) and performed time-lapse imaging every 15 minutes for 24 hours. The videos and all individual images were analyzed for the number of TNT connections and their cells of origin. The number of TNT connections between chemosensitive and chemoresistant cell lines and the cells initiating these connections varied depending on the cell lines used (Figure 3A and 3C). When chemoresistant (R) SKOV3 cells and chemosensitive (S) A2780 cells were co-cultured (Figure 3A, 3B), resistant cells formed significantly fewer TNTs to other resistant cells than did sensitive-to-sensitive cells (*p* = 0.001). No significant difference in TNT numbers was observed among sensitive-to-resistant cells as compared with resistant-to-sensitive cells (*p* = 0.298). When chemoresistant (R) C200 cells and chemosensitive (S) A2780 cells were co-cultured (Figure 3C, 3D), sensitive cells did not form significantly more TNTs to each other than did resistant cells (*p* = 0.437). However, in the SKOV3 and A2780 co-culture combinations (Figure 3A), sensitive cells formed significantly more TNTs to resistant cells than did resistant-to-sensitive cells (*p* < 0.0001). These results demonstrated variable, albeit reproducible, patterns in TNT formation among chemoresistant and sensitive cell lines.

### TNTs form between platinum-resistant ovarian cancer cells and benign ovarian epithelial cells *in vitro*

We had previously reported that TNTs are responsible for long-range intercellular transfer of cellular cargo, including oncogenic microRNAs, from malignant to benign and stromal cells [12]. In follow-up studies, we co-cultured the GFP-expressing platinum-resistant SKOV3 cells in a 1:1 ratio with benign ovarian epithelial (IOSE) cells stained with the red-fluorescing lipophilic dye DiI (Figure 3E, 3F). Cells were allowed to adhere and form TNTs among co-cultured cells. We again performed time-lapse imaging for 24 hours using inverted fluorescent microscopy to capture TNT formation between SKOV3 and IOSE cells (Figure 3F). The benign-to-benign and resistant-to-resistant interactions were equal to one another but greater than resistant-to-benign and benign-to-resistant connections. However, the differences between these possible TNT connections were not statistically significant (Figure 3E).

### Inhibition of mTOR disrupts TNT-mediated intercellular communication under normoxic and hypoxic conditions

**Figure 4:**
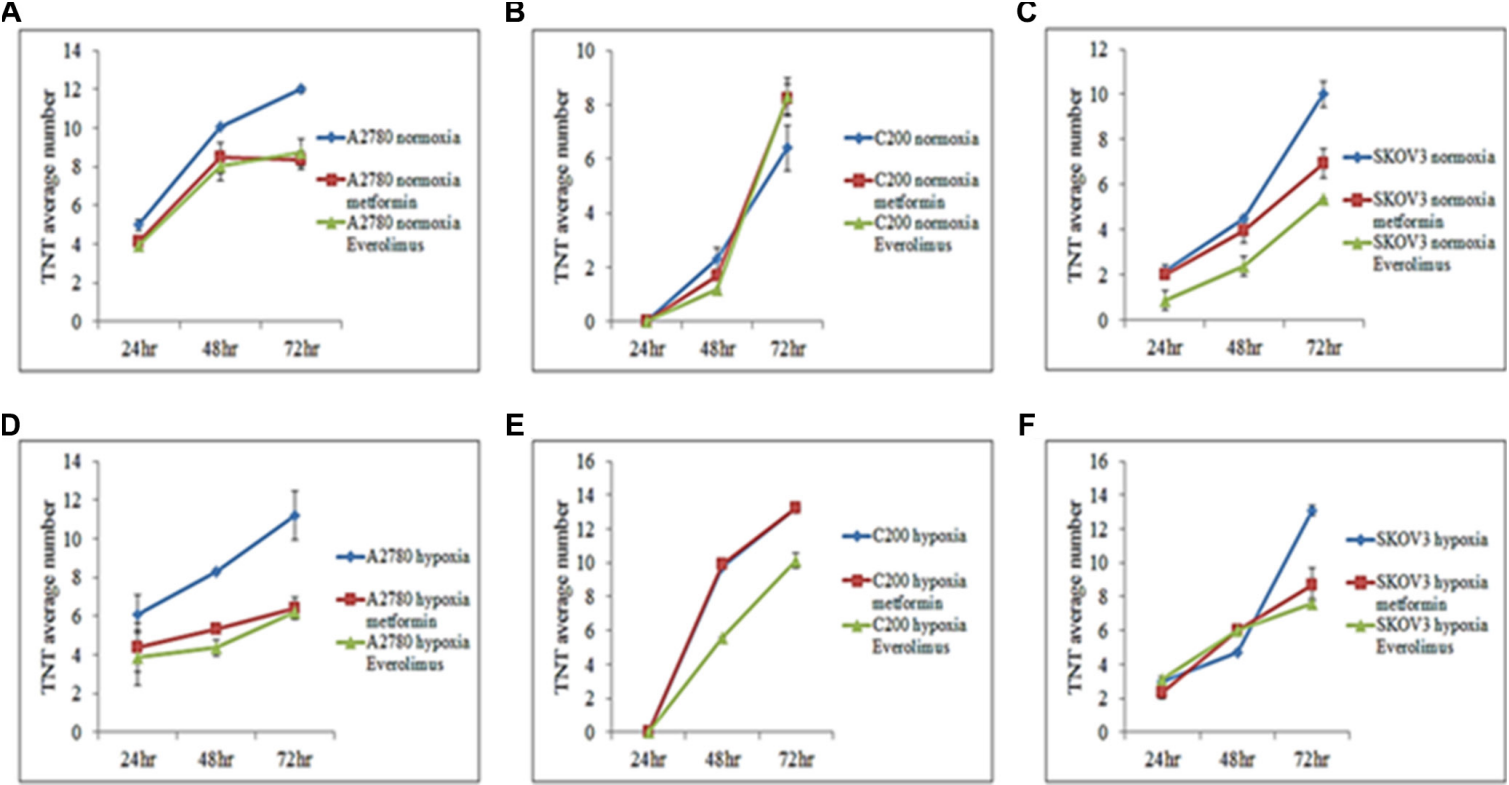
Effects of metformin and everolimus on TNT formation For all experiments, the number of TNTs in 10 high-power fields (hpf) were counted and averaged and each experiment was performed in duplicate. (**A**) Number of TNTs (mean ± standard deviation) in A2780 cells with or without addition of metformin or everolimus under normoxia. (**B**) Number of TNTs (mean ± standard deviation) in C200 cells with or without addition of metformin or everolimus under normoxia. (**C**) Number of TNTs (mean ± standard deviation) in SKOV3 cells with or without addition of metformin or everolimus under normoxia. (**D**) Number of TNTs (mean ± standard deviation) in A2780 cells with or without addition of metformin or everolimus under hypoxia. (**E**) Number of TNTs (mean ± standard deviation) in C200 cells with or without addition of metformin or everolimus under hypoxia. (**F**) Number of TNTs (mean ± standard deviation) in SKOV3 cells with or without addition of metformin or everolimus under hypoxic conditions.

mTOR inhibition suppresses TNT formation *in vitro* [10]. We determined whether mTOR inhibition could interfere with TNT formation in ovarian cancer cells. Everolimus is a clinically available direct inhibitor of mTOR, whereas metformin indirectly inhibits mTOR through activation of 5′ AMP-activated protein kinase (AMPK). We found that both metformin and everolimus appeared to decrease the number of TNTs in all A2780 and SKOV3, but not in C200 cells (Figure 4A–4C). Overall, everolimus was minimally more effective than metformin at reducing the number of TNTs. Interestingly, TNT formation was most dramatically inhibited in platinum-resistant SKOV3 as well as chemosensitive A2780 cells.

We also examined the effects of mTOR inhibition on TNT formation under hypoxic conditions in cultures of C200 cells in comparison with its parent cell line A2780. Both metformin and everolimus had similar effects in these cells under both hypoxic and normoxic conditions in A2780 and SKOV3 cell culture; however, only everolimus appeared effective in suppressing TNTs in C200 cells, but metformin did not (Figure 4D, 4E, 4F). It was interesting that mTOR inhibition dramatically reduced TNT formation in the chemosensitive A2780 cell line under hypoxic conditions, whereas it had much less of an effect on TNT formation in its derivative chemoresistant C200 cell line. This findings suggested alternative pathways for TNT formation and upregulation in chemoresistant ovarian cells compared with chemosensitive cells.

Suppression of the mTOR pathway is known to affect cellular proliferation [23]. Thus, we also examined the effects of everolimus and metformin on cell proliferation (Supplementary Figure 3). We used a standard CCK-8 assay to assess cell metabolism as a surrogate marker for proliferation rate. Metformin effectively suppressed cellular proliferation in both A2780 and C200 cells cultured in either a normoxic or hypoxic environment (Supplementary Figure 3). Everolimus effectively suppressed cell proliferation in both A2780 and C200 cells under normoxic conditions; however, under hypoxic conditions, the effect of this drug was attenuated in A2780 cells. Everolimus was ineffective in suppressing cell proliferation for C200 cells cultured in hypoxia (Supplementary Figure 3). These data support the notion that changes in the number of TNTs were independent of cell number and differences in cellular metabolism rate and represented absolute changes in TNT formation.

## DISCUSSION

**Figure 5:**
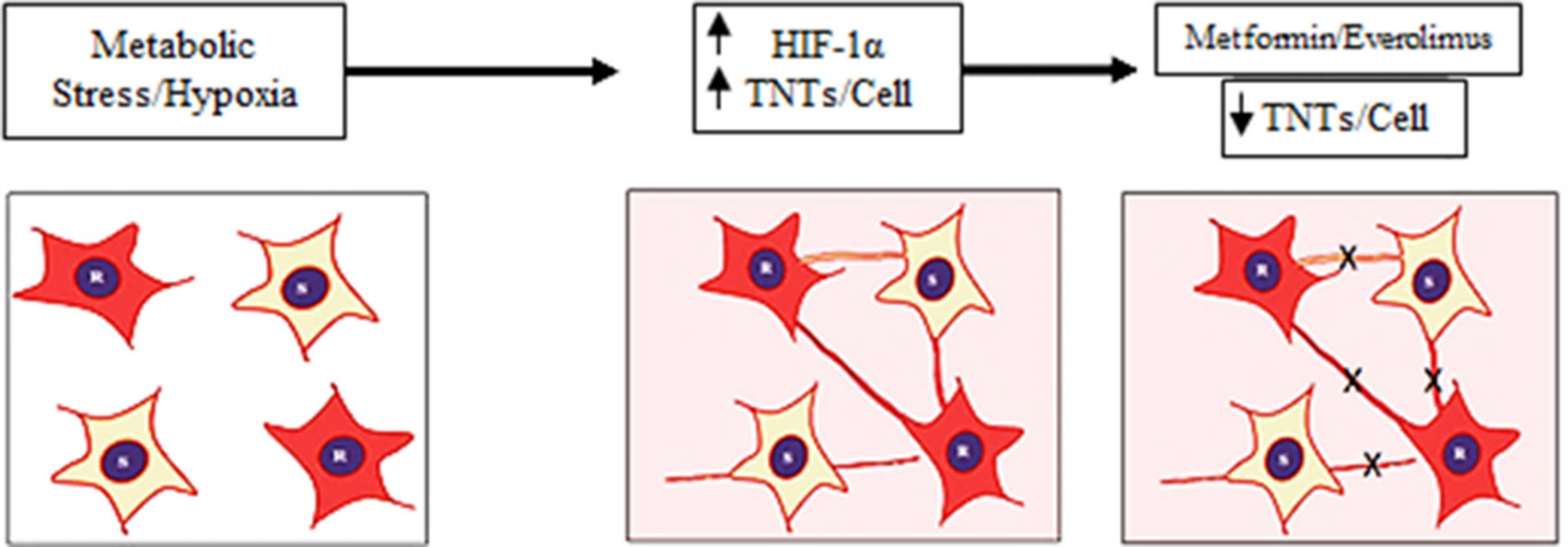
Summary diagram Physiologic stress induced under hypoxic conditions leads to an increase in HIF-1α expression and subsequent increase in TNTs. This increase in TNTs allows cells to form cellular networks that facilitate sharing of cellular signals. Inhibition of the mTOR pathway using clinically available drugs (everolimus, metformin) can suppress TNT-mediated intercellular communication.

In the present study, we focused on the clinically relevant problem of platinum chemotherapy resistance by assessing differences in formation between chemoresistant and chemosensitive ovarian cancer cells in the setting of hypoxia. We were able to reproducibly show TNT formation among chemoresistant and chemosensitive ovarian cancer cells. We concluded that there were quantifiable differences in TNT numbers between chemoresistant and chemosensitive malignant ovarian cells independent of differences in cellular proliferation. Our data suggest that both malignant and normal ovarian cell lines can serve as an *in vitro* model to assess the role of TNTs in ovarian cancer. Using chemoresistant ovarian cancer cells as a model, our data suggest that cellular stress in the form of hypoxia can also induce TNT-mediated communication and that this occurs at a higher rate in chemoresistant cells (Figure 5). The effectiveness of mTOR inhibition in suppressing TNT formation represents a potentially novel and complementary approach to cancer-directed therapy.

The emergence of resistance to standard-of-care chemotherapy drugs and biologic agents remains a major clinical problem, resulting in increased morbidity and mortality in cancer. This is especially true in those with high rates of recurrence, such as ovarian cancer. The molecular and cellular basis of chemoresistance is poorly understood and requires novel approaches for investigation. Common mechanisms may exist and be present across many forms of cancer. One example of a critical process responsible for tumor cell organization and heterogeneity that may explain development of chemoresistance is intercellular communication. Here we show that hypoxia-induced TNT formation is a novel mechanism of chemoresistance in cancer. In the past few years, *in vitro* studies have begun to elucidate the role of TNTs for facilitating intercellular trafficking of cellular signals between connected cancer cells [10, 12, 16, 24–26]. For example, our group has demonstrated *in vitro* direct cell-to-cell transfer of oncogenic genetic materials, including microRNAs, via TNTs connecting malignant ovarian cancer cells to each other as well as to stromal epithelial cells [10, 12, 16]. Furthermore, there is visual proof that TNTs exist in tumors from multiple cancer types imaged *ex vivo*, confirming that these structures are not *in vitro* artifacts, and may furthermore have a functional role *in vivo* [9, 10, 12]. The role of TNTs in cancer, as well as in other models of disease, represents a novel and important area of interest for understanding how long-distance cellular communication takes place in the complex and heterogeneous solid tumor microenvironment.

Intercellular transfer as a mechanism for ovarian cancer drug resistance specifically was proposed and studied more than 20 years ago [3]. These data suggested that metabolic cooperation between subsets of cells (chemoresistant and chemosensitive malignant ovarian cells) occurred to synchronize cells against drug therapy. TNTs have been proposed as a specific mode of communication responsible for causing and/or maintaining drug resistance [27]. Furthermore, the finding that cell-to-cell transfer of antibiotic-resistance genes via TNTs induces changes in recipient bacteria serves as a unique and highly pertinent parallel to TNT-mediated chemoresistance [28]. Studies which have demonstrated the propagation of signals for apoptosis, such as caspase proteins [29], via TNTs support the notion that other similar-sized signals could be transmitted as well. Together, these studies support the notion that there are multiple ways that TNTs are involved in the development of chemoresistance among refractory malignancies.

Our study addressed differences in interactions between specific chemoresistant and chemosensitive cells using ovarian cancer as an *in vitro* model system. It is likely that variability exists between different cancer cell types based on properties from site of origin, and also depending on resistance to specific drugs and the underlying mechanisms of that resistance (eg platinum resistance in our study, as compared to resistance to other classes of biologic or chemotherapeutic drugs). Our quantification of cell-cell interactions demonstrated that resistant-to-sensitive cell interactions made up only a minority of all interactions observed in co-cultures. However, if only a few interactions are needed for a chemoresistant cell to connect with a chemosensitive cell, that provides profound implications and insight into tumor cell behavior. Do chemoresistant cells efficiently connect to cells and transmit signals that could eliminate their “competition” in the tumor microenvironment by killing off cells that otherwise would use up valuable nutrients and prevent effective local cell invasion? If so, then this would represent a novel and powerful mechanism by which chemoresistant cancer cells survive.

If TNTs play a contributing biologic function by facilitating chemoresistance, then their targeting in a selective manner may represent a previously undiscovered approach to cancer therapy. Our finding that pharmacologic inhibition of the mTOR pathway decreased the rate of formation of TNTs between ovarian cancer cells may provide a rationale for a new approach to preventing development of chemotherapy resistance via a TNT-mediated mechanism.

Currently, it is well known that drugs used for *in vitro* studies, such as actin-destabilizing agents, can readily disrupt TNTs, but these agents also non-specifically disrupt other actin-based filaments as well, including intracytoplasmic actin and their stress fibers [10]. In our prior studies, we identified several compounds in clinical use or being investigated for treatment for cancer and other metabolic diseases that disrupt and/or suppress TNT formation in cancer. Although mTOR inhibitors are not selective for TNTs *per se*, inhibition of mTOR may also affect the cellular stress response and assembly of actin-based TNTs. This concept is supported by established data regarding the role of the mTORC2 complex in actin assembly and formation [30–32]. Further investigation is warranted regarding the role of the mTOR pathway as a metabolic impetus for TNT formation.

## MATERIALS AND METHODS

### Cell lines and cell culture

A2780, C200, SKOV3, and IOSE cell lines were kindly obtained from Dr. Sundaram Ramakrishnan at the University of Minnesota. The A2780 cell line is an ovarian tumor-derived epithelial cell line; the C200 cell line is derived from the A2780 line, which is resistant to 10 times the concentration of cisplatin. The SKOV3 cell line is derived from the ovarian adenocarcinoma ascites of an untreated patient. SKOV3 cells are resistant to cisplatin, TNF, diphtheria toxin, and doxorubicin [24]. IOSE cells are immortalized from normal ovarian epithelial cells. All cell lines were authenticated using sequence tandem repeat genotype profiling (Johns Hopkins University, STR Profiling for Human Cell Line Authentication) and confirmed by comparison to available genetic profiles using the University of Colorado database (website: https://www.dom.umn.edu/divisions/hematology-oncology-and-transplantation/lou-lab) [34]. A2780 and SKOV3 cell lines were passaged per protocol using RPMI-1640 media with 10% fetal bovine serum (FBS), 1% penicillin-streptomycin (P-S), 2% L-glutamine, and 0.002% plasmocin. The C200 cell line was cultured per protocol in RPMI-1640 media with 10% FBS, 1% P-S, 2% L-glutamine, 0.00025% insulin, and 0.002% plasmocin. The IOSE cell line was cultured per protocol in DMEM media with 10% FBS, 1% P-S, 2% L-glutamine, and 0.002% plasmocin. All cell lines were maintained at 5% CO_2_ and at 37°C. Cell lines were passaged every 2–3 days using trypsin-0.53 mM EDTA solution, kept in T-75 cm^2^ tissue culture flasks, and confirmed to be negative for mycoplasma infection.

### Procurement and imaging of nanotubes in tumors

Formalin-fixed primary ovarian tumor tissues and secondary metastatic tumors were obtained via the Tissue Procurement Facility at the University of Minnesota following Institutional Review Board (IRB) approval and patient consent. Preparation procedures and techniques for visualizing nanotubes in intact tumors have been previously published [10, 12]. Briefly, tumors were first fixed in 10% Neutral Buffered Formalin (Sigma, St Louis, MO) for at least 3 days. For visualization of nanotubes in intact human tumors, MitoTracker Red (250 nM), Hoechst 33342 (10 μg/ml), and phalloidin 2.5% were used to stain mitochondria, nuclei, and actin, respectively. Fixed tumor specimens were then washed with phosphate-buffered saline (PBS) three times and then cut into small slices 1.0 cm in length and 1.0−1.5 mm in thickness. Tumor slices were then washed again in PBS and stained with 500 nM solution of MitoTracker Orange CM-H2TMRos (M-7511, Life Technologies, Grand Island, NY) and 0.5 μg/ml Hoechst 33342 (Molecular Probes, Life Technologies, Grand Island, NY) in PBS for 10 minutes under cover to avoid exposure to light. Excess stain was removed by washing the slices several times with PBS. A stained tumor slice was mounted onto an Attofluor Cell Chamber (Cat A-7816, Life Technologies, Grand Island, NY) with a 25 mm round cover glass of 1.5 mm thickness (Warner Instruments, Hamden, CT) on both sides. Sections were mounted for use in the Nikon A1R-MP Multiphoton Confocal Microscope at the University of Minnesota Imaging Center. Stained tumor slices were imaged on the Nikon A1R-MP microscope using a PlanApo LWD 25× water-immersion lens. Imaging data were collected by z-stacked optical sectioning and then analyzed using NIS elements AR software (Nikon, version 4.00.07).

### Imaging of TNTs in cell lines

For visual staining of cell lines and primary malignant cells from effusions, all cells were cultured in T culture flasks and transferred to 6-well adherent tissue culture plates (Fisher Scientific, Pittsburgh, PA) at 37°C in 5% CO_2_ prior to imaging. Fluorescent lipophilic dyes DiI and DiO were used per manufacturer's instructions (Invitrogen).

### Culture conditions for TNT formation in normoxia and hypoxia

Cells were cultured in a low-serum, high-glucose environment to determine the growth of TNTs as previously described [10]. Briefly, the culture conditions consisted of RPMI-1640 medium with 2.5% FBS, 50 mM glucose, 1% P-S, 2% L-Glutamine, 10 nM ammonium lactate, and pH 6.6. For hypoxic conditions, cells were then placed in 10-cm cell culture plates and placed in a chamber containing 2% oxygen, 5% carbon dioxide, and 93% nitrogen.

### Quantification of TNTs

TNTs were identified as previously described [10, 12, 14, 16, 28, 33]. Briefly, these parameters included (i) lack of adherence to the substratum of tissue culture plates, including visualization of TNTs passing over adherent cells; (ii) TNTs connecting two cells or extending from one cell were counted if the width of the extension was estimated to be < 1000 nm; and (iii) a narrow base at the site of extrusion from the plasma membrane. Cellular extensions not clearly consistent with the above parameters were excluded. An Olympus IX70 inverted microscope (Olympus Corporation) with 20× objective lens was used to count the number of TNTs and cells in 10 randomly chosen fields of each 6-well plate at 24, 48, 72, and 96 hours. A single representative image of each field was taken at all time points in each well. Experiments were performed in duplicate for each cell line. To determine TNT formation, three ovarian cancer cell lines (A2780, C200, SKOV3) and one benign ovarian epithelial cell line (IOSE) were plated at a density of 4 × 10^4^ cells/well in 6-well adherent tissue culture plates (Fisher Scientific, Pittsburgh, PA) at 37°C in 5% CO_2_ with TNT-inducing medium. TNTs and cells were counted manually, and the TNT index was calculated as the number of TNTs per cell (TNTs/cell) using previously described methods [16]. The variation within each experiment was high, and therefore, measures from ten random fields were taken per experiment and averaged to calculate a more accurate measure for each experiment.

### Western blotting for HIF-1α

Cells were harvested by centrifugation and lysed in standard radioimmunoprecipitation assay (RIPA) buffer containing Roche cOmplete Mini EDTA-Free Buffer with 2 mM dithiothreitol (Biocompare, South San Francisco, CA) using four 3-second bursts of sonication (Fisher Scientific Sonic Dismembrator Model 100, ThermoFisher Scientific). Total protein was quantitated using the Bio-Rad Protein Assay (Bio-Rad, Hercules, CA; Cat#500-0006) and read on a Beckman DU-64 Spectrophotometer (Beckman Coulter, Brea, CA). Protein (60 μg) was resolved on a precast 10% Mini-Protean TGX 10-well gel (Bio-Rad, Hercules, CA; Cat#456-1033) at 100 V for 1 hour and transferred to a nitrocellulose membrane per the manufacturer's protocols (Bio-Rad, Hercules, CA; Cat#162-0097). The membrane was cut into two pieces (upper and lower) and blocked for 2 hours at room temperature in 5% milk made with Tris-buffered saline. After washing (3 × 5 minutes each, at room temp), the upper half of the membrane was incubated with anti-HIF-1α (clone-H1α67) mouse monoclonal primary antibody at 10 μg/ml (Sigma, Cat#H6536), and the lower half was incubated with mouse monoclonal anti-β actin (clone-AC15) at 1:10,000 dilution (Sigma, Cat#A5441). Both halves were incubated overnight at 4°C. The next day, membranes were washed and then incubated with horseradish peroxidase (HRP) conjugated goat anti-mouse IgG (H+L) secondary antibody (Pierce, Prod#31430) for 2 hours at room temperature. After another set of washings, detection was achieved using the SuperSignal West Dura Extended Duration Substrate (Pierce, Prod#34076) and exposed to Kodak Biomax Light Film (Kodak, Prod#178 8207) for 1 to 20 seconds for β-actin and overnight for HIF-1α, following standard protocols.

### Cell proliferation assays

Cell proliferation assays were performed using the Cell Counting Kit-8 (CCK8) per the manufacturer's instructions. Cells were plated at a density of 4,000 cells per well in a 96-well plate in triplicate with 200 μl of medium in each well. Cells were allowed to grow in standard conditions. 10 μl of CCK8 reagent was placed in each well at 4 time points (0, 24, 48, 72, and 96 hours) and left for incubation. Samples were then read in triplicate using a plate reader, and curves were created using Excel.

### Pharmacologic treatment of cell lines

A2780, C200, and SKOV3 cell lines were treated with metformin or everolimus. Concentrations of metformin (970 nM) and everolimus (40 μM) were based on previous studies of mesothelioma [10].

### Time-lapse imaging and quantification of cell-to-cell interactions

For A2780 and C200 cell interactions, C200–DiO (green) and A2780-DiI (red) cells in a ratio of 1:1 were plated simultaneously in 6-well plates at a density of 5 × 10^4^ cells/well. For A2780 and SKOV3 interactions, SKOV3– GFP (green) and A2780-DiI (red) cells in a ratio of 1:1 were plated in 6-well plates at a density of 5 × 10^4^ cells/well. Plates were evaluated after four hours to ensure even distribution of both cell lines. Cells were allowed 12 hours to settle and adhere to the plates prior to imaging. For time-lapsed imaging, multiple 20× fields of view containing evenly distributed cells were chosen using a wide-field Zeiss Axio200M microscope costume-fitted with a stage incubator that maintains environmental conditions at 37°C and 5% CO_2_. The microscope was set up to take an image of each chosen field every 12 minutes in the DIC (differential interference contrast) red and green fluorescent channels. Twenty-four hours of imaging from the start of each time-lapse was used for analyzing cell-to-cell interactions. TNT formation among co-cultured cells was identified to occur between resistant-to-resistant (R-to-R), sensitive-to-sensitive (S-to-S), resistance-to-sensitive (R-to-S), and sensitive-to- resistance (S-to-R) cells based on staining.

### Statistical analysis

Quantification of TNTs/cell was performed over a four-day period as described above. Overall comparisons of the medians for both TNTs/cell per field and cells per field across cell lines were analyzed using the Kruskal-Wallis test. For pairwise comparisons of TNT formation between resistant and sensitive cells, Wilcoxon Rank Sum two-sample tests were conducted and *p*-values were adjusted for multiple comparisons using a Bonferroni correction. For analyses with sample sizes less than 5, descriptive statistics were presented, however formal hypothesis testing was not performed. Analyses were conducted in SAS version 9.3 (Cary, NC), and *p*-values < 0.05 were considered statistically significant.

## CONCLUSIONS

Our results demonstrate that TNTs form *de novo* to connect chemosensitive and chemoresistant malignant ovarian cells and that chemoresistant cells are stimulated to communicate via TNTs under hypoxic conditions, whereas chemosensitive cells are not. Elucidating the molecular mechanisms and conditions that promote this process as well as identifying downstream molecular pathways affected by these changes is of critical importance.

## SUPPLEMENTARY MATERIAL FIGURES AND TABLES


