# Supplementary material for: Tunneling nanotube formation is stimulated by hypoxia in ovarian cancer cells
Source: Oncotarget. 2016 May 20;7(28):43150–61. doi: 10.18632/oncotarget.9504 (PMC5190014; doi:10.18632/oncotarget.9504)
Supplement: Supplementary file 1 [file oncotarget-07-43150-s001.pdf]

Tunneling nanotube formation is stimulated by hypoxia in ovarian cancer cells

Supplementary Materials

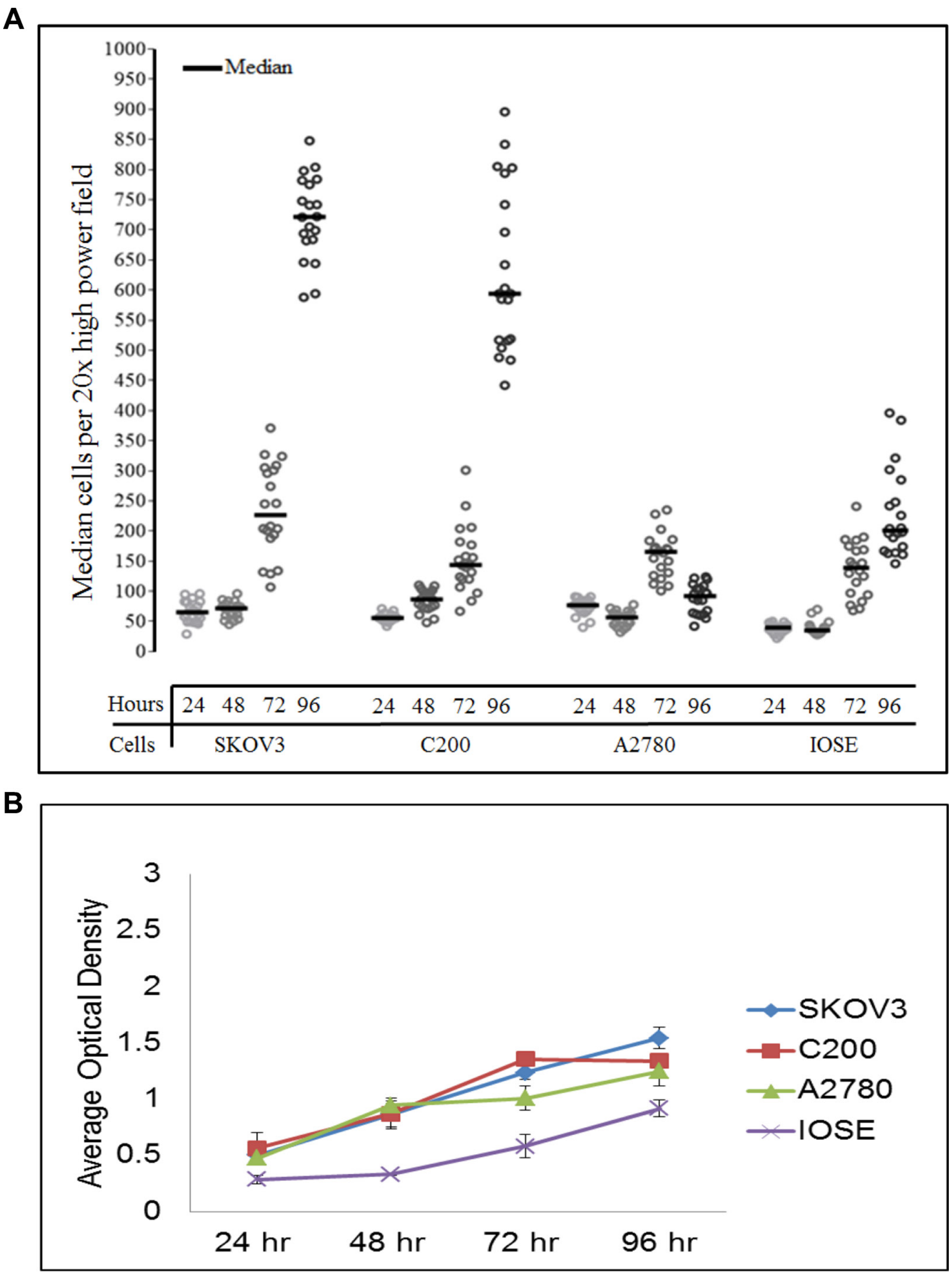

Supplementary Figure S1: Comparison of cellular proliferation among malignant and benign ovarian cells. (**A**) The number of cells per field in SKOV3, C200, A2780, and IOSE cell lines across replicates over the same four-day period are summarized using the median (line). An Olympus IX70 inverted microscope with 20x objective lens was used to visualize and count the number of TNTs and cells in 10 randomly chosen fields. (**B**) Optical densities (mean ± standard deviation) of SKOV3, C200, A2780, and IOSE cell lines after 24, 48, 72 and 96 hours. The Cell Counting Kit-8 (CCK-8) colorimetric assay was used as a surrogate marker for cell proliferation by measuring optical density at 450 nm. Each experiment was performed in duplicate.

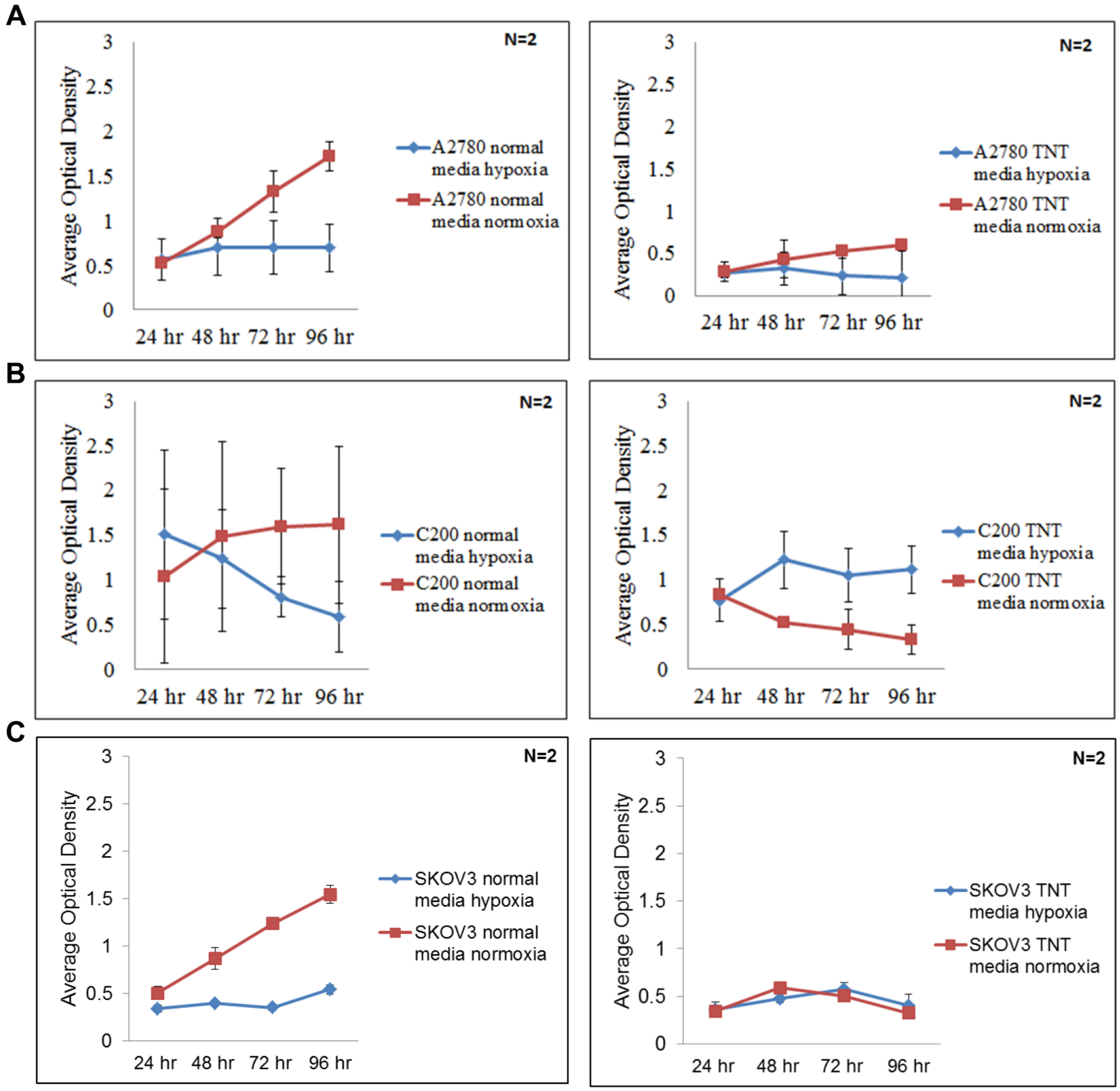

Supplementary Figure S2: Effects of hypoxia on cell proliferation of chemoresistant and chemosensitive ovarian cancer cells. Cells were seeded at equal densities in 96-well culture dishes under standard and TNT media in normoxic or hypoxic conditions. Normal medium (10% FCS, 25 mM glucose RPMI-1640) and TNT medium (2.5% FCS, 50 mM glucose RPMI-1640) are defined as described in Materials and Methods. After 24, 48, 72 and 96 hours, the Cell Counting Kit-8 colorimetric assay was used as a surrogate marker for cell proliferation by measuring optical density at 450 nm. Each experiment was performed in duplicate. (**A**) Optical densities (mean ± standard deviation) of A2780 chemosensitive cells under normoxic vs. hypoxic conditions, using standard culture medium (left panel) or TNT medium (right panel). (**B**) Optical densities (mean ± standard deviation) of C200 chemoresistant cells under normoxic vs. hypoxic conditions, using standard culture medium (left panel) or TNT medium (right panel). (**C**) Optical densities (mean ± standard deviation) of SKOV3 chemoresistant cells under normoxic vs. hypoxic conditions, using standard culture medium (left panel) or TNT medium (right panel).

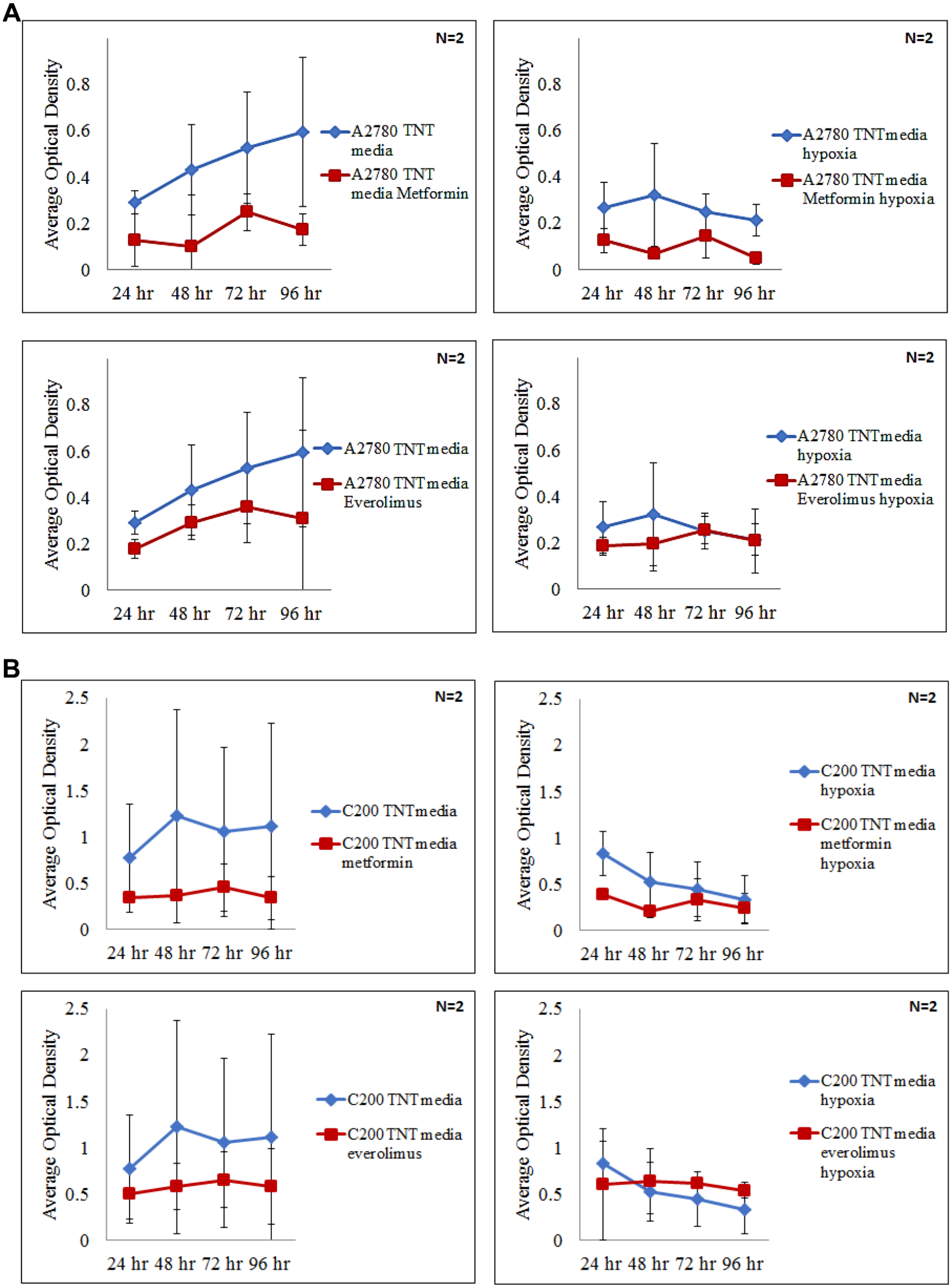

Supplementary Figure S3: Effects of metformin and everolimus on cell proliferation under normoxic or hypoxic conditions. Cells were grown in standard passage medium or in TNT medium under normoxic and hypoxic conditions and allowed to adhere to the tissue culture plate prior to treatment with the mTOR inhibitors everolimus or metformin. The CCK-8 assay was used as a surrogate marker of cell proliferation by measuring optical density as described in Supplemental Figure 1. (**A**) Optical densities (mean panels). (**B**) Optical densities (mean ± standard deviation) of chemoresistant C200 cells with and without treatment using metformin (top panels) or everolimus (bottom panels).

Supplementary Table S1: Statistical assessments comparing cell proliferation for each cell line used in this study

Cell Type	24 hours	48 hours	72 hours	96 hours	
	Median (Min, Max)	Median (Min, Max)	Median (Min, Max)	Median (Min, Max)	*p*-value over time
A2780	77 (40–91)	56.5 (32–78)	165.5 (101–235)	81 (42–124)	< 0.0001
C200	55.5 (42–71)	86.5 (48–110)	143.5 (67-301)	594 (442–896)	< 0.0001
IOSE	39.5 (22–50)	35 (28–70)	144 (68–241)	201 (146–396)	< 0.0001
SKOV3	65 (29–96)	71.5 (45–96)	226.5 (107–371)	721.5 (588–848)	< 0.0001
p-value across types	< 0.0001	< 0.0001	0.0002	< 0.0001	

The median numbers of cells are listed as shown with minimum/maximum values, as well as p-values across time and across cell types.

Supplementary Table S2: Statistical assessments of TNT index (number of TNTs/cell) for each cell line used in this study

Cell Type	24 hours	48 hours	72 hours	96 hours	
	Median (Min, Max)	Median (Min, Max)	Median (Min, Max)	Median (Min, Max)	*p*-value over time
A2780	0.01 (0–0.08)	0.05 (0–0.16)	0.12 (0.04–0.17)	0.10 (0–0.29)	< 0.0001
C200	0.02 (0–0.06)	0.02 (0–0.10)	0.03 (0–0.10)	0.02 (0–0.06)	0.078
IOSE	0.03 (0–0.13)	0.08 (0–0.27)	0.26 (0.13–0.47)	0.49 (0.08–1.12)	< 0.0001
SKOV3	0.04 (0–0.14)	0.03 (0–0.07)	0.01 (0–0.04)	0 (0–0)	< 0.0001
p-value across types	0.013	0.002	< 0.0001	< 0.0001	

The median numbers of TNTs/cell are listed as shown with minimum/maximum values, as well as p-values across time and across cell types.
